# Case Report: Concurrent left pyosalpinx and acute suppurative appendicitis after IVF-FET: a case initially misdiagnosed as heterotopic pregnancy

**DOI:** 10.3389/fmed.2026.1924108

**Published:** 2026-09-23

**Authors:** Dongyun Jia, Shuai Liu, Yang Chen, Xinyue Zhang

**Affiliations:** Center for Reproductive Medicine, The First Hospital of Jilin University, Changchun, Jilin, China

**Keywords:** acute appendicitis, gestational hypertensive disorders, heterotopic pregnancy, IVF-FET, pyosalpinx

## Abstract

**Background:**

Patients conceived via *in vitro* fertilization and frozen-thawed embryo transfer (IVF-FET) may develop atypical acute abdominal manifestations, which pose substantial diagnostic difficulties in early gestation.

**Case presentation:**

A 24-year-old pregnant woman after IVF-FET presented with persistent hypogastric pain, and transvaginal ultrasound identified an adnexal complex mass. Heterotopic pregnancy (HP) and ovarian torsion were top differential diagnoses, prompting emergency laparoscopic exploration. Intraoperative findings ruled out ectopic gestation and confirmed an extremely rare concurrent lesion: left pyosalpinx combined with acute suppurative appendicitis.

**Intervention & outcome:**

The patient received laparoscopic left salpingectomy, appendectomy and extensive pelvic adhesiolysis, accompanied by perioperative antibiotics and tocolytic agents. Postoperatively, she developed hypertensive disorders of pregnancy (HDP), which may have been associated with the preceding severe pelvic suppurative infection and its systemic inflammatory response, although a causal relationship cannot be established from a single case and IVF-FET itself is a known risk factor for HDP. She finally delivered a healthy male neonate by cesarean section at 37⁺⁴ weeks of gestation without neonatal adverse outcomes.

**Conclusion:**

Gestational pyosalpinx is a rare entity that shares overlapping clinical and sonographic features with heterotopic pregnancy, leading to frequent misdiagnosis. Timely laparoscopic surgery safely eliminates infectious lesions while maintaining intrauterine pregnancy, representing an effective therapeutic strategy for such complicated cases. The observed temporal association between severe pelvic infection and subsequent HDP warrants further investigation.

## 1 Introduction

Pregnancy complicated by pyosalpinx is an exceedingly rare and complex clinical entity that presents immense diagnostic and therapeutic challenges (1). Timely identification is often hindered because specific signs of pelvic inflammatory disease (PID) are frequently absent in these patients (2, 3). On routine transvaginal ultrasound, a pyosalpinx can easily be missed or mischaracterized; its imaging features, paired with symptoms like lower abdominal pain, closely mimic those of a hemorrhagic ectopic pregnancy—especially when a patient presents without a fever (4).

With the increasing utilization of assisted reproductive technologies and the popularization of IVF technology, complications have risen (5–7). When a complex adnexal mass and pelvic free fluid are detected in a woman who has undergone IVF with the transfer of two or more embryos, heterotopic pregnancy (HP) is typically the leading differential diagnosis, given its heightened incidence of approximately 1 in 100 in this population (8). Because the clinical presentation completely overlaps with the typical imaging manifestations of a ruptured heterotopic pregnancy, and because the patient often lacks a fever or typical peritoneal irritation signs, the underlying pelvic abscess can easily be masked (9). Both pyosalpinx in early pregnancy and HP carry fatal consequences for the intrauterine fetus, posing a high risk of pregnancy loss or severe maternal morbidity (10, 11).

What makes this specific case unique is its extraordinary anatomical distribution and clinical rarity. Anatomically, the appendix is adjacent to the right fallopian tube; thus, severe acute appendicitis may directly spread infection to the right adnexa and trigger a right-sided pyosalpinx (12). In contrast, a concurrent infection involving appendicitis and a left-sided pyosalpinx is exceptionally rare because the appendix and the left adnexa are not anatomically adjacent. To our knowledge, no relevant cases of left-sided suppurative pyosalpinx complicated with appendicitis following assisted reproductive technology have been reported in the published literature. This case represents, to our knowledge, the first reported instance of a left-sided pyosalpinx co-occurring with acute suppurative appendicitis in a pregnant woman following IVF-FET.

Furthermore, the concurrent presentation of left-sided pyosalpinx and acute suppurative appendicitis in an early IVF-FET pregnancy represents a particularly rare clinical entity with significant diagnostic challenges. Both conditions can manifest with atypical or overlapping features in gestation, and their coexistence complicates preoperative differentiation from more common complications such as heterotopic pregnancy or hemorrhagic ovarian cysts. Herein, we report this case to highlight the diagnostic pitfalls and therapeutic considerations in managing complex pelvic infections following assisted reproductive technology.

## 2 Case description

### 2.1 Patient information

The patient is a de-identified 24-year-old female (gravida 0, para 0, abortus 0) with a history of primary infertility secondary to polycystic ovary syndrome (PCOS) and male-factor teratozoospermia. She had no prior history of PID, pelvic surgeries, or relevant chronic medical conditions.

### 2.2 Past interventions and outcomes

#### 2.2.1 Pre-IVF workup

A hysterosalpingography performed one year prior to IVF demonstrated bilateral patency of the fallopian tubes.

#### 2.2.2 Oocyte retrieval

Controlled ovarian hyperstimulation (COH) was initiated using a 14-day protocol of letrozole and human menopausal gonadotropin (HMG). Routine ultrasounds during tracking showed completely normal fallopian tubes. Preoperative complete blood counts (CBC) and vaginal secretion analyses were unremarkable. Following vaginal irrigation and sterile transvaginal oocyte retrieval, 11 oocytes were collected. Fresh embryo transfer was canceled to mitigate the risk of ovarian hyperstimulation syndrome (OHSS). Cleavage-stage embryos (*n* = 4) and one blastocyst were successfully cryopreserved. Oral cefuroxime axetil (250 mg twice daily for three days) was administered post-retrieval, and she experienced no symptoms of pelvic infection.

#### 2.2.3 Embryo transfer (FET)

Three months later, a hormone replacement cycle was prepared. When her endometrial thickness reached 7.5 mm, two day-3 frozen embryos were transferred. Pre-FET screening (CBC and vaginal secretions) was completely normal, and no signs of infection were reported.

#### 2.2.4 Early pregnancy course

A serum hCG test confirmed pregnancy 9 days post-FET. An intrauterine anechoic area was identified at 15 days post-FET. At 21 days post-FET, she experienced transient, mild lower abdominal pain and minor vaginal bleeding which resolved spontaneously without medical intervention.

### 2.3 Physical examination & clinical findings upon presentation

At 25 days post-FET (approximately 6 weeks of gestation), the patient presented for a routine evaluation where transvaginal ultrasound confirmed a viable clinical intrauterine pregnancy (gestational sac measuring 1.8 * 2.7 * 1.4 cm, a yolk sac, and a 0.6 cm embryo with positive cardiac activity) (Figure 1A). However, the scan also revealed concerning adnexal findings:

**Figure 1:**
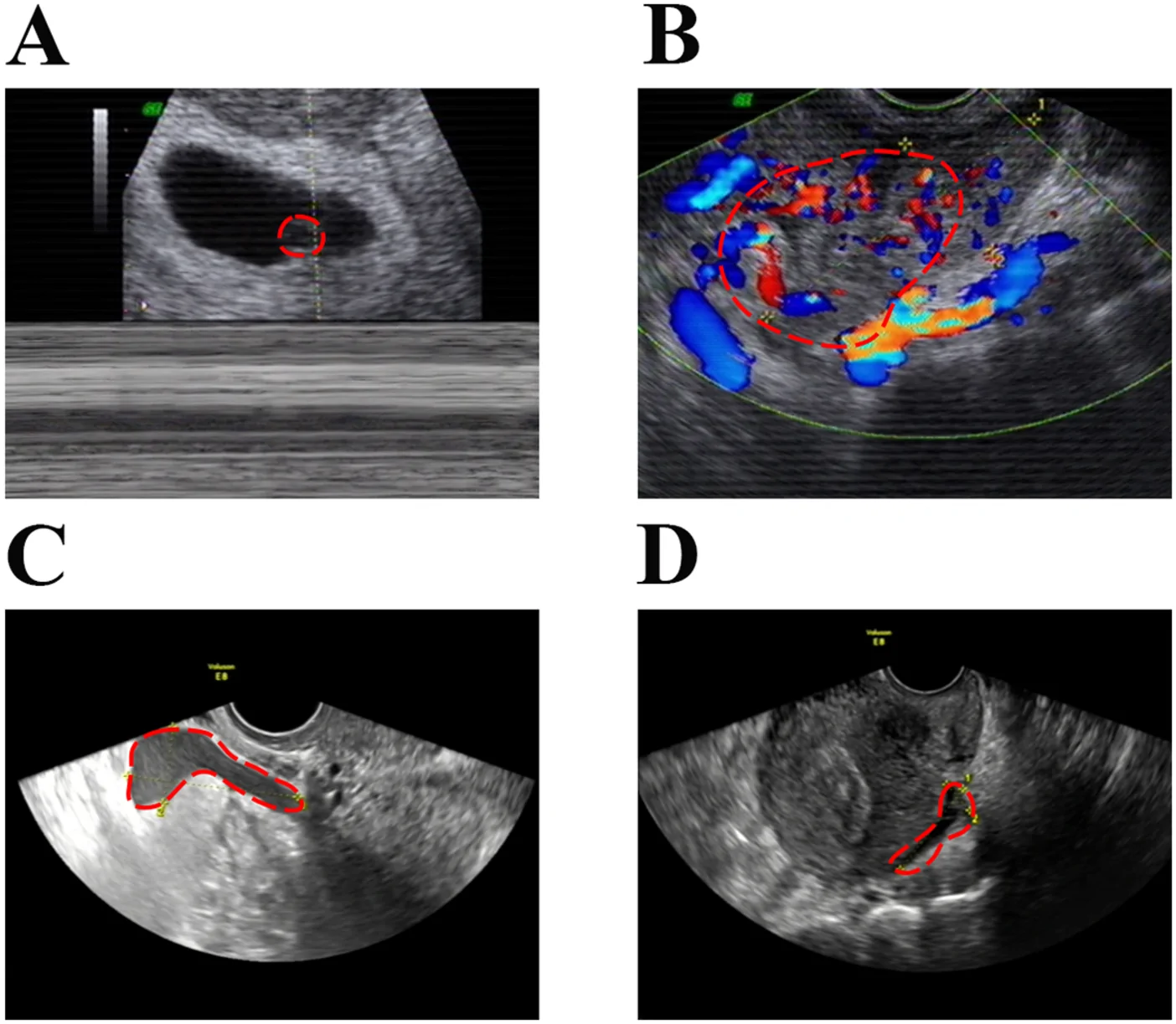
**(A)** Yolk sac with detectable cardiac activity within the uterine cavity. **(B)** Heterogeneous echogenic area adjacent to the left ovary. **(C)** Free fluid anterior to the uterus. **(D)** Free fluid posterior to the uterus.

A heterogeneous, highly vascular, echogenic area measuring 5.3*2.2 cm adjacent to the left ovary (Figure 1B).

Free fluid measuring 4.5 *2.0 cm in the anterior cul-de-sac (Figure 1C).

Free fluid measuring 3.3* 1.1 cm in the posterior cul-de-sac (Figure 1D).

The patient was immediately hospitalized under suspicion of a hemorrhagic pelvic cyst, adnexal torsion, or a heterotopic pregnancy. Upon admission, her vital signs were:

**Body Temperature:** 36.5 °C (afebrile)
**Heart Rate:** 128 beats per minute (tachycardia)
**Blood Pressure:** 100/80 mmHg
**Respiratory Rate:** 16 breaths per minute.

Abdominal examination revealed mild tenderness localized to the mid-lower abdomen. Notably, there was a complete absence of rebound tenderness, muscle guarding, or classic right lower quadrant localized pain. Laboratory investigations revealed a mild leukocytosis (WBC count: 11.27 *10^9^). Considering the viable intrauterine pregnancy and after communicating with the patient, invasive diagnostic procedures such as culdocentesis and radiation-exposing examinations like abdominal CT scans were not performed immediately.

## 3 Timeline

The chronological clinical care timeline for this patient is summarized in Table 1.

**Table 1:** Chronological clinical care timeline for the IVF-FET pregnancy complicated by left pyosalpinx and suppurative appendicitis.

Time node	Clinical events	Imaging/laboratory results	Clinical intervention & decision
Pre-IVF	Hysterosalpingography	Bilateral fallopian tubes patent	Plan COH and oocyte retrieval
Oocyte retrieval cycle	Ovulation induction, egg collection, antibiotic prophylaxis	11 oocytes retrieved, no infection signs	Cancel fresh embryo transfer to prevent OHSS
FET Day 0	Two day-3 embryos transferred	Endometrium 7.5 mm, normal vaginal secretion	Discharge after routine transfer observation
FET Day 9	Biochemical pregnancy	Serum hCG positive	Regular pregnancy monitoring arranged
FET Day 21	Spontaneous lower abdominal pain + slight vaginal bleeding	No emergency examination performed	Patient refused hospital visit, symptoms self-resolved
FET Day 25	Confirmed intrauterine clinical pregnancy; incidental left adnexal mass and pelvic free fluid	Intrauterine embryo with cardiac activity; left ovarian heterogeneous mass, bilateral cul-de-sac effusion	Admitted for observation, suspected heterotopic pregnancy/ovarian torsion
Admission day (approximately 6 weeks’ gestation)	Stable vital signs, mild lower abdominal tenderness, elevated neutrophils	WBC 11.27 × 10⁹/L, no fever; CT and culdocentesis avoided for fetal safety	Emergency laparoscopic exploration scheduled
Intraoperative laparoscopy (approximately 6 weeks’ gestation)	Extensive pelvic adhesion, left fallopian tube torsion with pus, suppurative appendicitis, no ectopic mass	Purulent exudate in pelvic cavity	Pelvic adhesion lysis, left salpingectomy, appendectomy, pelvic drainage placement
Postoperative day 1	Transient leukocytosis, peak temperature 37 °C	WBC peak 22.71 × 10⁹/L, wound drainage unobstructed	Uterine contraction inhibition + perioperative broad-spectrum antibiotics
Postoperative course	Intravenous antibiotic and tocolytic therapy	Aerobic and anaerobic cultures of pelvic fluid negative	Cefuroxime sodium 0.75 g IV q8 h for 2 days; atosiban 6.75 mg IV bolus, then 18 mg/h for 3 h, then 6 mg/h for 45 h
Postoperative pathology	Left chronic suppurative salpingitis, pyosalpinx, appendiceal serosal necrosis with suppurative inflammation	Aerobic & anaerobic culture of pelvic fluid negative	Confirm diagnosis of combined pyosalpinx + appendicitis
32 weeks gestation	New-onset hypertension, diagnosed HDP	BP 150/94 mmHg and 146/95 mmHg (4 h apart); trace proteinuria; platelets 198 × 10⁹/L; ALT 28 U/L; AST 24 U/L; creatinine 62 μmol/L; BUN 4.8 mmol/L	Oral labetalol 100 mg three times daily started
Gestation 37⁺⁴ weeks	Cesarean delivery	Live male infant, birth weight 3,485 g, length 49 cm, no neonatal complications	Delivery for HDP in the setting of IVF-conceived pregnancy
Postpartum	Blood pressure normalized within 2 weeks after delivery	–	Regular postpartum blood pressure monitoring

## 4 Diagnostic assessment, therapeutic interventions, follow-up and outcomes

### 4.1 Diagnostic assessment

The initial preoperative diagnosis leaned strongly toward HP or a hemorrhagic pelvic cyst, heavily influenced by the patient's history of a dual embryo transfer and the mixed-echogenicity vascular adnexal mass on Doppler ultrasound. Definitively ruling out pyosalpinx or appendicitis was impossible prior to surgery due to the complete lack of a fever, absence of classic right lower quadrant tenderness, and the decision to forgo CT imaging to avoid fetal radiation in this clinical setting.

While abdominal CT scanning is not absolutely contraindicated in pregnancy and may be used with appropriate shielding when clinically justified, it was deferred in this case. Non-contrast pelvic magnetic resonance imaging (MRI), which lacks ionizing radiation and offers high sensitivity and specificity for appendicitis in pregnant patients, could have been considered to reduce diagnostic ambiguity. However, given the patient's hemodynamic status (tachycardia of 128 bpm), the ultrasound findings suggestive of a vascular adnexal mass with hemoperitoneum-like free fluid, and the high clinical suspicion of a potentially urgent surgical condition such as ruptured heterotopic pregnancy or adnexal torsion, immediate diagnostic laparoscopy was prioritized over additional imaging.

### 4.2 Therapeutic intervention

Given the patient's severe abdominal pain, tachycardia of 128 beats per minute, and undifferentiated fluid collections anterior and posterior to the uterus on ultrasonography, an emergency exploratory laparoscopy was performed on the day of admission at approximately 6 weeks’ gestation. Intraoperatively, extensive and dense pelvic adhesions were observed among the bowel, uterus, bilateral fallopian tubes, and ovaries. The left fallopian tube was densely adhered to the retroperitoneum, partially enveloped by the left ovary, and complicated by tubal torsion. A large amount of thick, yellow-brown purulent exudate accumulated in the pelvic cavity, and a markedly congested appendix encased by adjacent bowel loops was also identified.

A colorectal surgeon was called intraoperatively to perform an appendectomy and complex pelvic/intestinal adhesion lysis. Exploration confirmed the absence of an ectopic gestational sac; however, both tubes showed chronic inflammatory edema, and the left tube was filled with frank pus, confirming a left-sided pyosalpinx. A definitive left-sided salpingectomy was performed to eradicate the source for sepsis development (Figure 2A). Drainage tubes were positioned in both the left adnexal region and the appendiceal bed. Histopathological evaluation later confirmed pyosalpinx, chronic suppurative salpingitis, and tissue necrosis localized to the appendiceal serosa (Figure 2B). Based on the postoperative pathological report and the intraoperative findings, the clinical diagnosis was acute suppurative appendicitis.

**Figure 2:**
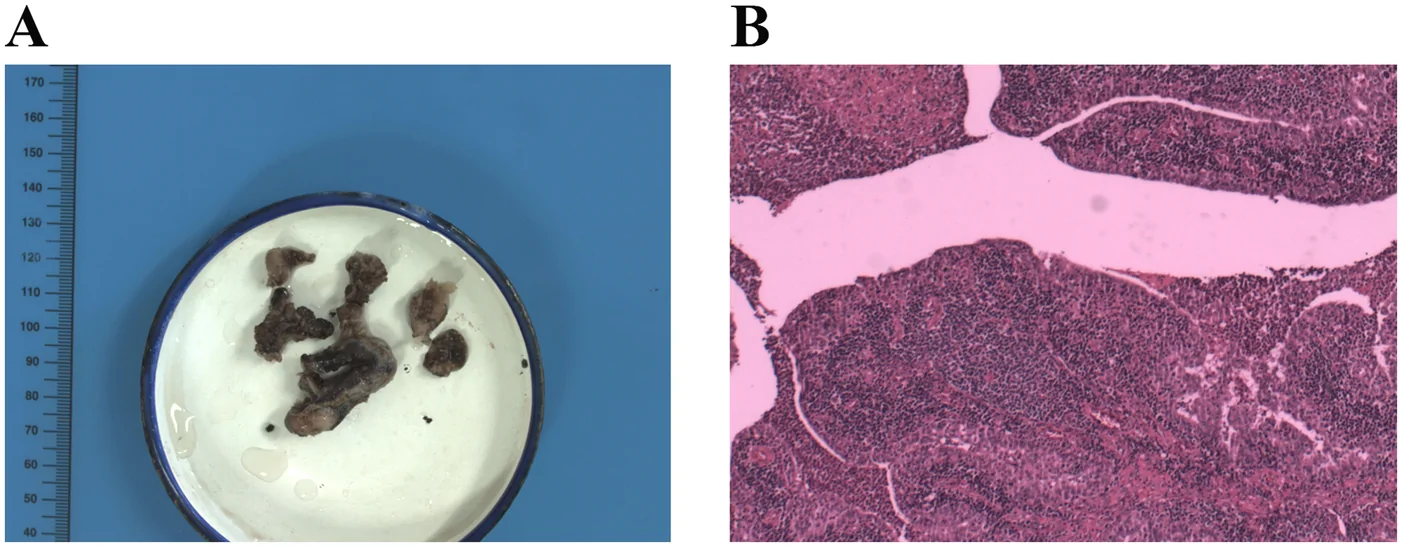
**(A)** The left salpinx. **(B)** Microscopic view of the left fallopian tube following pathological staining, demonstrating dense inflammatory cell infiltration.

Perioperative antimicrobial management consisted of cefuroxime sodium 0.75 g administered intravenously every 8 h for 2 days. Postoperatively, tocolytic therapy with atosiban was initiated with a single rapid intravenous bolus of 6.75 mg, followed by a continuous intravenous infusion at 18 mg/h for 3 h and then at 6 mg/h for 45 h to reduce uterine contractility. Although the intrauterine pregnancy was ultimately preserved, this favorable outcome likely reflects the combined effects of infection source control, antibiotic therapy, and reduced uterine irritability, and cannot be attributed solely to tocolysis. The total duration of hospitalization for the initial admission was three days, and postoperative intravenous antibiotics were administered for two days.

### 4.3 Follow-up and outcomes

Aerobic and anaerobic cultures of the aspirated pelvic fluid yielded no bacterial growth. Postoperatively, the patient was managed with intravenous antibiotics and tocolytic therapy, as detailed above.

During subsequent antenatal care, the patient developed new-onset hypertension at 32 weeks’ gestation. Blood pressure was 150/94 mmHg and 146/95 mmHg on two separate occasions four hours apart. Urinalysis showed trace protein. Laboratory evaluation revealed a platelet count of 198 × 10⁹/L, alanine aminotransferase 28 U/L, aspartate aminotransferase 24 U/L, serum creatinine 62 μmol/L, and blood urea nitrogen 4.8 mmol/L. Oral labetalol 100 mg three times daily was initiated for blood pressure control.

At 37 weeks and 4 days’ gestation, she underwent cesarean delivery due to HDP in the setting of an IVF-conceived pregnancy and delivered a healthy, viable infant.

Postpartum blood pressure monitoring showed gradual normalization of hypertension within two weeks after delivery.

## 5 Discussion

Diagnosing pelvic infections during pregnancy is immensely difficult due to overlapping presentation profiles (13, 14). While transvaginal ultrasound boasts a high specificity (97%–100%) for pyosalpinx, its sensitivity remains low (32%–85%), meaning a negative scan cannot definitively rule out the condition. The ultrasound presentation of a vascular, mixed-echogenicity mass alongside free pelvic fluid in our patient strongly mirrored an intra-abdominal hemorrhage from a ruptured tubal pregnancy. Combined with the 1% incidence rate of heterotopic pregnancies following multi-embryo IVF transfers, clinicians almost universally mistake these infectious masses for ectopic tissue (15).

Furthermore, the mechanical pathogenesis in this case is highly unusual. Literature shows that severe acute appendicitis typically triggers right-sided salpingitis via direct, contiguous pathogen spread across the short anatomical distance. A left-sided pyosalpinx presenting concurrently with appendiceal serosal necrosis, without an isolated right-sided tubal abscess, is an extreme clinical rarity.

The pathological findings demonstrated appendiceal serosal necrosis with suppurative inflammation. Whether this represents primary acute appendicitis with secondary pelvic spread or secondary serosal inflammation from the generalized pelvic suppurative process cannot be definitively determined from the available evidence. The absence of a clearly identified transmural neutrophilic infiltrate typical of primary acute appendicitis suggests that secondary involvement should be considered as an alternative explanation.

However, it is critical to distinguish temporal association from causation. A single case report cannot establish a causal relationship. Moreover, IVF-FET pregnancies carry an independently increased risk of HDP compared with spontaneous conceptions (5). Our patient lacked classic risk factors for preeclampsia (such as an elevated BMI, history of chronic hypertension, or renal disease), which raises the hypothesis that the severe pelvic inflammatory episode may have contributed to her HDP. Nevertheless, her HDP may have developed independently or been influenced by multiple interacting factors. This observation is hypothesis-generating and warrants investigation in larger cohorts, rather than representing a mechanistic conclusion.

### 5.1 Strengths and limitations of this case management approach

Strengths: The primary strength of this case management was the swift decision to utilize laparoscopy rather than managed expectation or invasive needle aspiration (culdocentesis). Laparoscopy provided definitive dual utility as the gold-standard diagnostic tool and a safe therapeutic medium, allowing for immediate intervention by a multidisciplinary surgical team (gynecologists and a colorectal surgeon). Performing a complete salpingectomy directly eliminated the purulent reservoir, preventing toxic tubal fluid from draining into the uterine cavity and causing a septic abortion.

Limitations: The primary limitation was the lack of advanced preoperative imaging. Considering the radiation risks of abdominal CT, pelvic MRI—which features high sensitivity and specificity for identifying appendicitis in pregnant individuals without ionizing radiation—could have significantly reduced diagnostic ambiguity prior to incisions. Additionally, the exact direction of causality between the appendiceal and tubal inflammatory processes cannot be determined from the presented evidence.

Core take-away lessons from this case: Maintain Broad Differentials: Pyosalpinx and appendicitis should remain on the differential checklist for pregnant IVF patients presenting with an adnexal mass, even if typical markers like fever or localized lower-quadrant pain are entirely absent. Laparoscopy is Safe: Early laparoscopic salpingectomy and appendectomy are highly effective during the first trimester to safely clear pelvic infections while successfully safeguarding an intrauterine fetus. Inflammation Links to HDP: Severe pelvic inflammatory episodes during early pregnancy may represent a potential risk factor or contributing factor for subsequent HDP, though causation remains unproven and further research is needed.

## 6 Conclusion

Pyosalpinx during pregnancy is rare. Diagnosis is challenging in asymptomatic patients. Laparoscopic salpingectomy is a safe and effective management option for pyosalpinx in pregnancy. Compared with medical therapy, salpingectomy completely removes the infected fallopian tube and prevents ongoing purulent discharge into the uterine cavity. The HDP observed in this patient may have been associated with inflammation secondary to pyosalpinx, although causation cannot be established from a single case and IVF-FET itself confers an increased risk of HDP. The potential association between HDP and severe pelvic infection in pregnant patients warrants further investigation. As appendiceal necrosis was confined to the serosa without clear evidence of transmural inflammation, the possibility of secondary serosal involvement from pelvic infection should be considered alongside that of primary appendicitis. Management of acute appendicitis in pregnancy primarily involves early appendectomy, and outcomes following appendectomy are significantly more favorable during the first trimester.

## 7 Patient perspective

I had been trying to conceive for a long time before we finally succeeded with IVF. When I began experiencing pain in my lower abdomen during early pregnancy, I was extremely frightened. I thought it might be an ectopic pregnancy or that I would lose the baby. The doctors told me I needed emergency surgery, and I was very worried about whether the baby could survive the procedure. After waking up from surgery, I was so relieved to hear that the baby was still viable and had a strong heartbeat. Later, when my blood pressure rose at 32 weeks’ gestation, I was surprised because I had never had blood pressure problems before. However, the doctors took excellent care of me and adjusted my medication appropriately. Delivering a healthy baby boy at 37 weeks’ gestation made me feel that everything we went through was worthwhile. I would tell other women facing similar challenges: do not wait if you experience pain or bleeding during early pregnancy—go to the hospital immediately, follow your doctors’ advice closely, and try to stay strong. Timely treatment truly can save both mother and child.

## Data Availability

The original contributions presented in the study are included in the article/Supplementary Material, further inquiries can be directed to the corresponding author.
